# Type 2 diabetic mellitus related osteoporosis: focusing on ferroptosis

**DOI:** 10.1186/s12967-024-05191-x

**Published:** 2024-04-30

**Authors:** Yili Chen, Wen Zhao, An Hu, Shi Lin, Ping Chen, Bing Yang, Zhirong Fan, Ji Qi, Wenhui Zhang, Huanhuan Gao, Xiubing Yu, Haiyun Chen, Luyuan Chen, Haizhou Wang

**Affiliations:** 1 Guangdong Provincial Hospital of Traditional Chinese Medicine, Guangzhou University of Chinese Medicine, Guangzhou, 510006 China; 2 Guangzhou University of Traditional Chinese Medicine, Guangzhou, 510006 China; 3 Stomatology Center, Shenzhen Hospital, Southern Medical University, Shenzhen, Guangdong 510086 China

**Keywords:** Type 2 diabetes mellitus, Osteoporosis, Diabetic osteoporosis, Ferroptosis, Reactive oxygen species

## Abstract

With the aging global population, type 2 diabetes mellitus (T2DM) and osteoporosis(OP) are becoming increasingly prevalent. Diabetic osteoporosis (DOP) is a metabolic bone disorder characterized by abnormal bone tissue structure and reduced bone strength in patients with diabetes. Studies have revealed a close association among diabetes, increased fracture risk, and disturbances in iron metabolism. This review explores the concept of ferroptosis, a non-apoptotic cell death process dependent on intracellular iron, focusing on its role in DOP. Iron-dependent lipid peroxidation, particularly impacting pancreatic β-cells, osteoblasts (OBs) and osteoclasts (OCs), contributes to DOP. The intricate interplay between iron dysregulation, which comprises deficiency and overload, and DOP has been discussed, emphasizing how excessive iron accumulation triggers ferroptosis in DOP. This concise overview highlights the need to understand the complex relationship between T2DM and OP, particularly ferroptosis. This review aimed to elucidate the pathogenesis of ferroptosis in DOP and provide a prospective for future research targeting interventions in the field of ferroptosis.

**Supplementary Information:**

The online version contains supplementary material available at 10.1186/s12967-024-05191-x.

## Introduction

As the global population ages, two common conditions, namely diabetes mellitus (DM) and osteoporosis (OP), become increasingly prevalent. The 10th edition of the IDF Diabetes Atlas estimates that the prevalence of diabetes in the global population aged 20–79 years is estimated to be 10.5% (536.6 million) by the 2021, and will rise to 12.2% (783.2 million) by 2045 [1]. In China, approximately 30% of the elderly population (aged 60 years and older) had DM in 2020, with over 95% being T2DM [2]. Furthermore, the WHO estimated that more than 200 million women worldwide have OP [3]. In China, an epidemiological survey on OP revealed a prevalence rate of 19.2% among people aged 50 or above, with 32.1% in females and 6.9% in males. In those aged ≥ 65 years, the prevalence rate of OP was 32%, with 51.6% in females and 10.7% in males [4]. Globally, OP causes more than 8.9 million fractures annually, resulting in an osteoporotic fracture every 3 s [5]. One in three women aged > 50 years will experience osteoporotic fractures, as will one in five men aged > 50 years [6]. Osteoporotic fractures (fragility fractures) often lead to severe complications due to minor trauma (equivalent to falling from standing height or lower) [7].

Diabetic osteoporosis (DOP) is a systemic chronic metabolic bone disease characterized by abnormal bone tissue structure and reduced bone strength in patients [8]. Over 90,000 osteoporotic fractures occur globally each year, most of which are associated with DOP and pose a significant threat to human health and socioeconomic factors [5]. As glucolipid homeostasis is disrupted in DM, the unique diabetic microenvironment is characterized by abnormally increased levels of extracellular metabolites. It is well known that OP is one of the common complications of DM. According to related data, approximately 50–66% of patients with DM have decreased bone mineral density (BMD), and approximately 33% of them have been diagnosed with DOP. Over the past decade, extensive investigations of the diabetic microenvironment and mineral homeostasis have identified increased cortical porosity, imbalanced bone metabolism, and distorted bone microarchitecture as the three main characteristics of DOP [9–10]. Current studies indicate that DM, whether type I or type II, is a contributing factor to increased fracture risk [11], therefore, it is crucial to pay special attention to DOP, particularly in a large population of patients with T2DM. Studies have shown that ferroptosis plays an important role in the development and progression of T2DM and complications [12]. To identify a potential treatment approach and its clinical applications for this larger colony type 2 Diabetic osteoporosis (T2DOP). Therefore, there is a need to further elucidate the relationship between T2DM and OP by focusing on ferroptosis [13].

Ferroptosis is a form of apoptosis that relies on intracellular iron [14]. It is characterized by iron-dependent lipid peroxidation. The depletion of cellular glutathione (GSH) and reduced activity of glutathione peroxidase 4 (GPX4) results in the inability of GPX4 to catalyze the reduction of lipid peroxides in ferroptosis. This leads to the Fe2^+^ oxidation of lipids through the Fenton reaction, causing the accumulation of reactive oxygen species (ROS) and ultimately promoting cell ferroptosis [15]. Iron deposition in pancreatic β-cells accelerating diabetes progression [16]. Iron-related oxidative processes and ROS accumulation in pancreatic β-cells are closely associated with insulin resistance and insufficient insulin secretion, leading to the pathogenesis of T2DM [17, 18]. Disturbances in iron metabolism, including iron deficiency and overload, are also strongly associated with OP [19, 20]. Excessive iron accumulation mediated by NADPH oxidase 4 (NOX4) can cause iron metabolism in osteoblasts (OBs), leading to OP [21]. DOP progression is accompanied by impaired glucolipid homeostasis and elevated plasma glucolipid metabolites; hence, ferroptosis may play a vital role in DOP pathogenesis [13]. In this review, we comprehensively discuss the research progress of ferroptosis in T2DOP.

## Ferroptosis

**Fig. 1:**
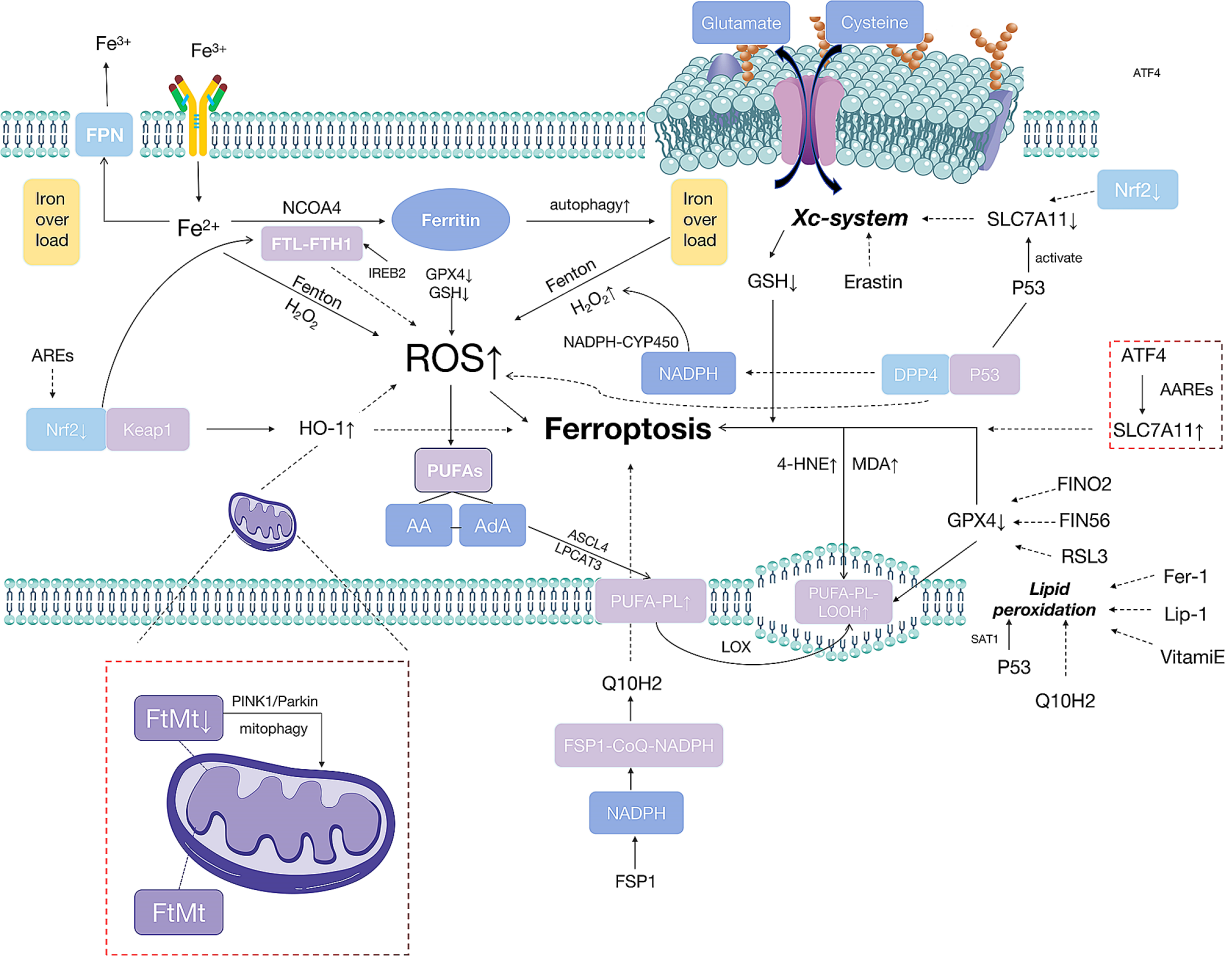
Map of ferroptosis

Ferroptosis is a novel iron-dependent form of programmed cell death (PCD), that is distinct from apoptosis, necrosis, and autophagy [14]. Morphologically, ferroptosis exhibits the following characteristics: (1) cells appear smaller and more rounded, with increased intercellular spacing; (2) the cell membrane remains intact, but there is cytoplasmic hemorrhage and no nuclear condensation; and (3) the number of mitochondria is reduced, with reduced or absent cristae, increased mitochondrial membrane density, and a ruptured outer mitochondrial membrane. Biological features include decreased cystine uptake, decreased GSH consumption, inhibition of cystine/glutamate reverse transport system (Xc system) activity, and abnormal accumulation of iron ions and ROS [22]. The most common lethal factors triggering ferroptosis are intracellular iron overload and excessive lipid peroxidation [23, 24]. And there is a simple map that makes ferroptosis easy to understand (shown in Fig. 1).

Iron overload is a critical factor for ferroptosis. Excess intracellular iron is exported by ferroportin (FPN) to maintain intracellular iron homeostasis [15]. Overloaded Fe2^+^ interacts with H_2_O_2_ via the Fenton reaction, leading to lipid peroxidation of polyunsaturated fatty acids (PUFAs) ultimately inducing ferroptosis [25]. Additionaly, resarch suggests that excessive autophagy and lysosomal activity promote ferroptosis through iron accumulation and lipid peroxidation. Ferritin (FTH) is a complex of ferritin light chain (FTL) and ferritin heavy chain 1 (FTH1). The degradation of FTH is dependent on autophagy and is termed ferritinophagy [26]. The Fe2 + stored in FTH can also be released into the cytoplasm. Nuclear receptor coactivator 4 (NCOA4) is a selective autophagy receptor that binds to FTH1 and mediates the delivery of intracellular ferritin to autophagosomes, ultimately releasing free iron [23]. Bellellelli et al. have proposed that iron overload initiates iron autophagy, which ultimately triggers ferroptosis [27]. Therefore, NCOA4 levels are key determinants of FTH engulfment flux, which is closely relatedd to ferroptosis.

Lipid peroxidation is thought to be a major factor in ferroptosis beacase lipid peroxides can destabilize cell membranes, thereby promoting membrane rupture [28]. Studies have shown that PUFAs, particularly arachidonic acid (AA) and adrenergic acid (AdA), are highly sensitive to lipid peroxidation and readily react with ROS, leading to lipid peroxidation and ultimately triggering ferroptosis [29]. Ferroptosis cells accumulate oxidized phospholipids, particularly phosphatidylinositol (PL) [30]. Phosphatidylethanolamine (PE), containing AA or AdA, is a critical PL that induces ferroptosis. Excessive ROS damage cellular components, including DNA, because lipid peroxidation directly damages phospholipids in cell and organelle membranes [31]. Acyl-CoA synthetase long-chain family member 4 (ACSL4) is a pivotal enzyme in lipid metabolism. ACSL4 catalyzes the synthesis of long-chain polyunsaturated-CoAs with a preference for AA and AdA, thus facilitating their esterification into phospholipids. PUFA-CoA is then transformed into phospholipid PUFAs (PUFA-PL) through the action of lysophosphatidylcholine acyltransferase 3 (LPCAT3) [32] and subsequently undergoes oxidation by lipoxygenases (LOXs) to produce lipid peroxides (LOOH), ultimately inducing cell ferroptosis [23]. Furthermore, the sensitivity of PUFA to lipid peroxidation is associated with the presence of highly reactive hydrogen atoms in the methylene bridges. Hydroxyl radicals interact directly with PUFA in phospholipid membranes, influencing the formation of lipid peroxides and triggering ferroptosis [33]. Products of lipid peroxidation, such as nucleic acids and proteins, as well as derivatives like 4-Hydroxynonenal (4-HNE) and malondialdehyde (MDA), can damage cells. These derivatives can be used as markers for ferroptosis and lipid peroxidation [34]. Nicotinamide adenine dinucleotide phosphate (NADPH) - cytochrome P450 (CYP450) reductase transfers electrons from NADPH to oxygen, resulting in hydrogen peroxide (H_2_O_2_) production, which then reacts with Fe. This generates hydroxyl radicals that disrupt the integrity of cell membranes by causing peroxidation of PUFA chains in membrane phospholipids [35]. Additionally, ferroptosis promotes LOX catalysis, leading to the generation of lipid peroxides in cell membrane phospholipids, which further enhances ferroptosis. FINO2 oxidizes ferrous iron to generate ROS and lipids to produce lipid peroxides [36].

GPX4 is a glutathione-related peroxidase enzyme [37] closely associated with ferroptosis. GSH is a tripeptide synthesized from cysteine, glutamic acid, and glycine [38]. With the catalytic action of selenium-containing cysteine (the active site of GPX4), GSH can reduce LOOH to harmless lipid alcohols [39]. Therefore, selenium deficiency in the serum or cytoplasm may impair GPX4 activity. The depletion of intracellular GSH and decreased GPX4 activity prevent the metabolic reduction of lipid peroxides catalyzed by GPX4, leading to the accumulation of ROS and ultimately promoting ferroptosis [15]. Studies have shown that RAS-selective lethal small molecule 3 (RSL3) contains a chloroacetamide moiety that reacts with nucleophilic amino acid residues on GPX4, resulting in GPX4 inactivation. Thus, RSL3 induces ferroptosis [40]. Ferroptosis inducing (FIN56) depletes coenzyme Q10 (CoQ10), degradates GPX4 and induces cellular ferroptosis, whereas 1,2-dioxin cycloalkane (FINO2) leads to iron oxidation and loss of GPX4 enzyme activity. FIN56 interferes with the mevalonate pathway. It affects the homeostasis of selenium proteins and other antioxidant molecules, which are essential for balancing OCs and OBs [41–43].

Additionally, the inhibition of the Xc system can further promote ferroptosis. The Xc system mediates a 1:1 exchange of intracellular glutamate for extracellular cysteine, reducing cysteine inside the cell to synthesize the antioxidant GSH [44]. Small-molecule compounds such as erastin reduce the synthesis of cysteine-dependent reduced GSH by inhibiting the Xc system, leading to intracellular GSH depletion, thereby causing the accumulation of a large number of peroxides and triggering cell ferroptosis. SLC7A11 is a light-chain subunit and a reverse transport protein that comprises the Xc system [45]. The SLC7A11 gene’s flanking region contains a site that perfectly matches the binding sequence of P53 which is a critical tumor suppressor gene. Thus, SLC7A11 is a downstream target of P53’s downstream targets [46–47]. P53 activation significantly reduces the expression of SLC7A11, which inhibits the Xc system and promotes ferroptosis [46]. Furthermore, P53 enhances the expression of metabolism-related genes, including spermidine/spermine N1-acetyltransferase 1 (SAT1), a transcriptional target of P53, leading to lipid peroxidation [48]. Research has revealed that multiple antioxidant systems can counteract ferroptosis [49].

(1) Iron responsive element binding protein 2 (IREB2) is an RNA-binding protein that regulates iron homeostasis. It significantly increases the expression of iron metabolism-related genes such as FTH1 and FTL, thereby inhibiting ferroptosis [28].

(2) P53 plays a dual role in regulating ferroptosis [47]. When P53 binds to dipeptidylpeptidase-4 (DPP4) in the nucleus, it inhibits NADPH oxidase and reduces ROS levels, thereby inhibiting ferroptosis [50].

(3) Nuclear factor E2-related factor 2 (Nrf2) is a vital transcription factor that regulates the transcription of antioxidant enzymes [51–52]. Under physiological conditions, Nrf2 interacts with Kelch-like ECH-associated protein 1 (Keap1) in the cytoplasm. Keap1 mediates Nrf2 ubiquitination and degradation, maintaining low levels of Nrf2f [53]. Nrf2 activity is tightly regulated by Keap1, a ubiquitin ligase adaptor protein for Cullin3-based E3 ligase. It isolates Nrf2 frpm the cytoplasm, preventing Nrf2 from entering the nucleus, and exerts its transcriptional activity to coordinate the expression of cell-protective genes, thereby maintaining cellular homeostasis. Even when a small amount of free Nrf2 moves to the nucleus, it binds to antioxidant response elements (AREs) and mediates the expression of basal ARE-dependent genes to maintain intracellular homeostasis [54–55]. Nrf2 regulates downstream gene transcription and prevents lipid peroxidation and ferroptosis by inducing the synthesis of genes such as SLC7A11 [56]. Keap1-Nrf2 prevents ferroptosis by upregulating the heme oxygenase-1 (HO-1) and FTH1 pathways [57].

(4) Activating transcription factor 4 (ATF4) promotes the transcription of SLC7A11 by binding to amino acid reaction element (AARE) in the SLC7A11 promoter region [58]. ATF4 can inhibit ferroptosis by activating SLC7A11 expression [59].

(5) Dihydroorotate dehydrogenase (DHODH) is a flavin-dependent enzyme located in the mitochondrial inner membrane, catalyzing the fourth step of pyrimidine nucleotide synthesis [60]. DHODH suppresses mitochondrial iron apoptosis by regulating the production of the coenzyme QH2 (CoQH2) in the inner mitochondrial membrane [61].

(6) Mitochondrial ferritin (FtMt) stores iron in mitochondria and protects them from iron-induced oxidative stress [62].

(7) Ferroptosis inhibitory protein 1 (FSP1) is an NADPH-dependent redox enzyme with DNA-binding activity [63]. FSP1 reduces CoQ10 to coenzyme Q10H2 (CoQ10H2) via its redox enzyme activity. CoQ10H2 is a lipophilic, free radical-scavenging antioxidant (RTA). The FSP1-CoQ-NAD(P)H axis inhibits ferroptosis by catalyzing the continuous regeneration of CoQ10 [64].

(8) The mevalonate pathway is a protein isoprenylationpathway. Mevalonate regulates the maturation of selenocysteine-specific tRNA (Trsp), which is required for the synthesis of selenium enzymes, including GPX4. Inhibition of the mevalonate pathway downregulates Trsp and GPX4 synthesis [65]. Recent research has indicated that FSP1 has an antiferroptotic mechanism parallel to the cysteine-GSH-GPX4 axis. FSP1 reduces CoQ10 to decrease lipid radicals, inhibit lipid peroxidation and subsequently ferroptosis [66].

(9) Additionally, lipid peroxidation can be inhibited by ferrostatin-1 (Fer-1), liproxtatin-1 (Lip-1), and vitamin E, which are free radical scavengers that can reduce lipid peroxidation and effectively block ferroptosis [67–68].

## Ferroptosis in OP

**Fig. 2:**
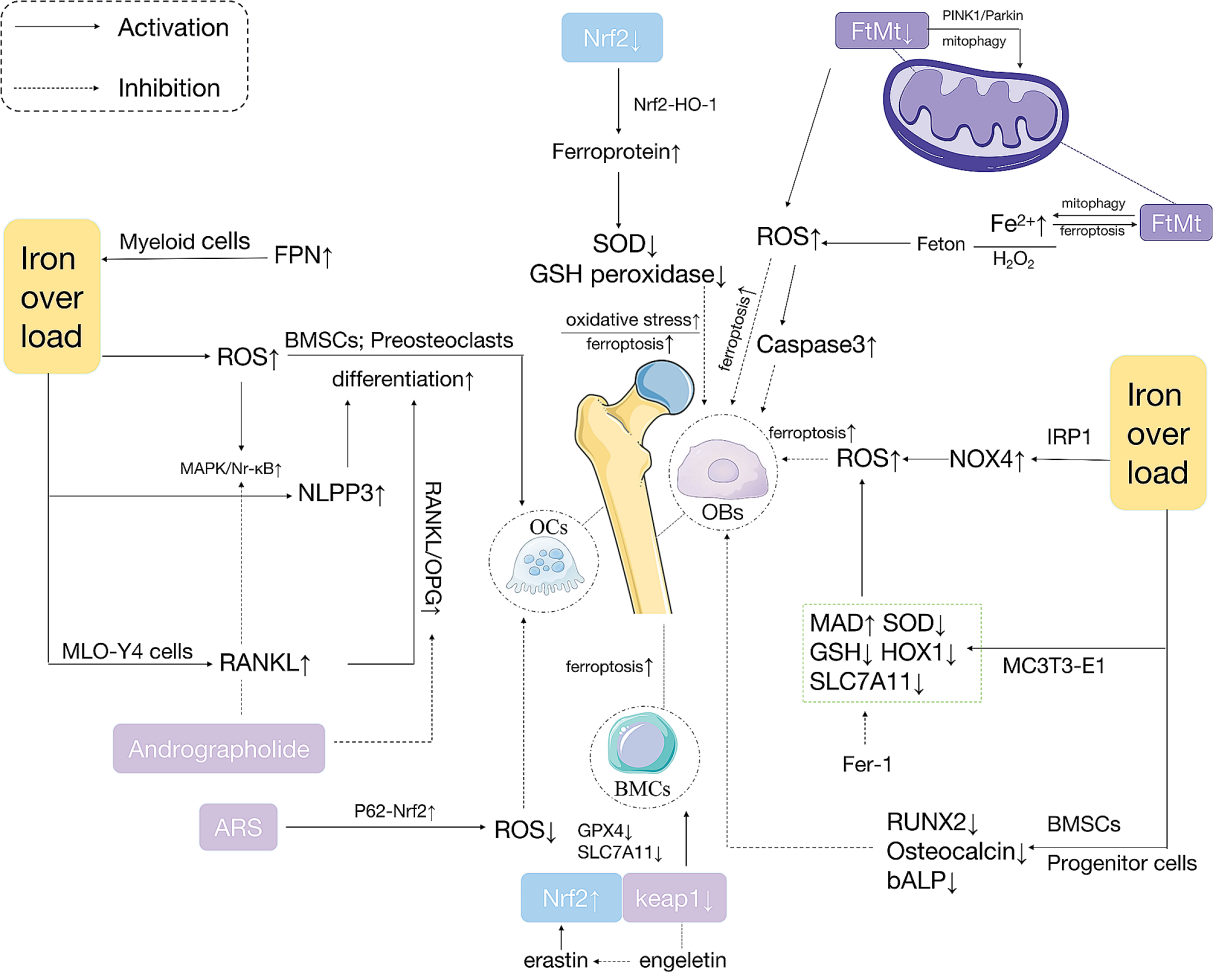
Osteoporotic ferroptosis

OP is a systemic disorder of bone metabolism characterized by reduced bone mass and structural damage, resulting in increased bone fragility and susceptibility to fracture [69]. The pathophysiology of OP involves a dynamic imbalance between bone resorption and formation, resulting in reduced bone mass and the destruction of trabecular bone, which are closely linked to OBs and Osteoclasts (OCs) [70]. Studies have reported that disturbances in iron metabolism, encompassing both iron deficiency and iron overload, may result in a dynamic imbalance between bone resorption and formation, contributing to OP [20, 71]. Excess Fe is considered toxic in animal models. Mice with iron overload exhibit increased oxidative stress, characterized by elevated ROS levels, and develop OP [72]. Some clinical studies have suggested that appropriate iron chelation therapy can prevent OP [73]. Maintaining iron homeostasis and reducing the levels of ROS can effectively prevent OP. Substantial evidence suggests that the oxidative stress induced by iron overload contributes to the pathophysiology of OP [44]. Consequently, the mechanisms through which iron overload leads to osteoporotic ferroptosis have received increasing attention (shown in Fig. 2).

### Ferroptosis in BMSCs

The source of OBs is the bone marrow mesenchymal stem cells (BMSCs), which exert their regulatory functions through remodeling. Iron overload-mediated oxidative stress, which increases ROS levels, is toxic to BMSCs, impairs mitochondrial dysfunction, and reduces their mineralization and osteogenic differentiation ability of BMSCs [74].

Nrf2/Keap1 pathway has been found to be involved in the oxidative stress and inflammatory response. Erastin-induced ferroptosis causes an increase in Nrf2 and a decrease in Keap1 expression in BMSCs, with increased expression of TFR1/FPN1 and decreased expression of GPX4 and SLC7A11. Engeletin (dihydrokaempferol 3-rhamnoside), an effective endogenous antioxidant, inhibits erastin-induced ferroptosis in BMSCs via the Nrf2/Keap1 pathway [75].

The NOP2/Sun RNA methyltransferase 5 (NSUN5) -FTH1/FTL pathway could be a causative event leading to ferroptosis of BMSCs. NSUN5 Recruits TRAP1 to Modify FTH1 and FTL. NSUN5 is downregulated in erastin-induced BMSC ferroptosis by increasing FTH1 and FTL levels, which inhibit ferroptosis [76].

### Ferroptosis in OBs

OBs are derived from BMSCs, and their bone formation plays an important role in maintaining the stability of bone structure and balance of bone metabolism [77]. Iron overload can affectt the OBs in various ways.

#### Iron overload has an inhibitory effect on the differentiation of OBs

(1) Iron overload suppressed OB differentiation. Runt-related transcription factor 2 (Runx2) is a necessary transcription factor for OB differentiation and maturation [78]. Iron overload can downregulate the expression of Runx2 and its targets, such as alkaline phosphatase and osteocalcin, in BMSCs and osteoprogenitor cells, thereby inhibiting OB formation [79].

(2) Additionally, the use of deferoxamine, an iron chelator, or superparamagnetic iron oxide nanoparticles with anti-osteogenic properties, reversed the inhibitory effect of free iron on BMSC differentiation into OBs [80].

#### Iron overload can affect the activity and function of bone cells

(1) Elevated NOX4 levels have been observed in the osteoporotic mouse and human bone tissues. Iron can directly interact with iron regulatory protein 1 (IRP1) in OBs, leading to IRP1 separation from iron-responsive element (IRE)-like sequences within the NOX4 gene. This promotes the expression of NOX4, resulting in increased ROS production when NADPH is used as a substrate. Accumulation of lipid peroxides causes mitochondrial dysfunction and promotes iron-dependent cell death in OBs [21].

(2) Protection through the Nrf2/HO-1 pathway: Activation of the NRF2/HO-1 pathway can significantly reduce ferritin levels. NRF2 activation initiates cellular defense mechanisms by increasing the expression of antioxidant enzymes, including GPX and superoxide dismutase(SOD). The toxic effects of iron overload on OBs are reduced by reducing oxidative stress, thereby inhibiting ferroptosis and promoting bone growth [81–83].

(3) The redox homeostasis of MC3T3-E1 mouse embryonic osteoblasts can be disrupted by iron overload, as manifested by excess intercellular ROS, lipid peroxidation, increased MDA, and decreased SOD and GSH, whereas Fer-1 improves the antioxidant homeostasis of the above peroxides. Iron overload inhibits the protein expression of GPX4, and haem oxygenase 1 (HMOX1), and increases the protein expression of FTH. Iron overload inhibits the osteogenic differentiation and mineralization of MC3T3-E1 cells [84]. Furthermore, iron overload impaired the viability of MC3T3-E1 pre-OB cells and induce apoptosis [85].

(4) Ammonium ferric citrate (FAC) significantly suppresses OBs function, inhibit ferroptosis, and activate the ASK1-p38 pathway, which is associated with anti-osteogenic effects [86]. Ferroptosis in OBs under high glucose/high fat conditions may be associated with the methyltransferase-like 3 (METTL3)/ASK1-p38 pathway. In an OB model, treatment of pre-OB cells (MC3T3-E1) with high glucose and palmitic acid (HGPA) will not only inhibit OBs differentiation and mineralisation, but also induce OBs ferroptosis [87]. Iron overload disrupt OBs activity and function by inducing ferroptosis via the ASK1/p38 pathway.

(5) Abnormalities in iron metabolism can lead to the accumulation of highly reductive free Fe^2+^ in OBs, causing oxidative stress. Excess Fe^2+^ ions can generate significant amounts of hydroxyl radicals and other ROS via Fenton reactions. ROS can stimulate the release of cytochrome C from mitochondria and activate apoptotic proteins such as caspase3, ultimately leading to apoptosis in OBs. ROS scavengers, such as N-acetylcysteine (NAC) can restore OBs activity [88–89].

(6) Mitochondrial ferritin (FtMt) is a protein that stores ferrous ions in cellular mitochondria. FtMt inhibits the onset of ferroptosis in OBs by reducing oxidative stress induced by excess ferrous ions [63]. FtMt deficiency induces mitochondrial autophagy by activating the ROS/PINK1/Parkin pathway, resulting in the release of more free Fe2 + from the mitochondria, which also occurs through the Fenton reaction and causes OBs ferroptosis [25].

Some studies have shown that inhibiting ferroptosis in OBs through the Nrf2/GPX4 pathway can improve bone formation capacity and microstructure. Vitamin D receptor (VDR) activators (1,25(OH)2D3) suppress ferroptosis in OBs by activating the Nrf2/GPX4 pathway [90]. Melatonin can improve bone microstructure both in vivo and in vitro by inhibiting ferroptosis in OBs. Melatonin reduces ROS levels, increases SLC7A11 levels, and enhances GPX4 activity by opening the NRF2/HO-1 antioxidant pathway, thereby reducing the toxicity of lipid peroxides to protect the biomembrane system and inhibit ferroptosis in OBs [81].

### Ferroptosis in OCs

OCs are terminally differentiated multinucleated cells of the monocyte/macrophage lineage with the unique function of resorbing bone matrix [91]. OCs play an important role in bone development and remodeling. The inhibition of bone resorption by OCs is essential for promoting bone matrix formation and bone remodeling [92]. Iron overload may also affecte OCs activity and funtion of OCs. Evidence suggests that FTN autophagy occurs when cells are iron deficient, making them more susceptible to intracellular Fe^2+^-induced ferroptosis [62, 93]. In addition, mature OCs require more cytoplasmic iron than the other cells. Therefore, OCs are more susceptible to ferroptosis [94]. OC formation and differentiation are associated with significant changes in the cellular iron homeostasis [95–96].

(1) A recent study showed that the lack of FPN in myeloid cells leads to iron accumulation and stimulates OC differentiation in mice. Iron overload induces ROS production and promotes OC differentiation [97].

(2) Macrophage-colony stimulating factor (M-CSF) and the receptor activator of nuclear factor κB ligand (RANKL) drive differentiation of mononuclear macrophage lineage cells and BMSCs, which later fuse to form multinucleated cells and further differentiate into mature OCs [98]. Iron overload significantly increases RANKL expression in MLO-Y4 cells (a mouse bone-like cell line), disrupted the RANKL/osteoprotegerin (OPG) balance, and promotes OC differentiation and bone resorption. Iron overload also induces apoptosis in MLO-Y4 and primary bone cells [99].

(3) ROS induced by iron overload has been reported to activate the mitogen-activated protein kinase (MAPK) pathway, which promotes osteoblast differentiation in bone metabolism and resorption [100]. Iron overload induces ROS accumulation, which activates the intracellular MAPK/NrκB/nucleotide-binding oligomerized structural domains, leucine-rich repeat sequences, and pyrin structural domains of 3 (NLRP3) signaling pathway, leading to OCs-mediated bone loss in OP [100].

Studies have shown that OP can be ameliorated by inhibiting the OCs. Artesunate (ARS) compounds inhibit iron uptake-stimulated OCs differentiation by inhibiting iron uptake, leading to ferroptosis [71]. ARS compounds inhibit ROS production and suppress ferroptosis during OCs differentiation by activating p62/Nrf2 signaling [100]. Andrographolide modulates OPG/RANKL signaling and inhibits the TNFα-activated NFκB pathway, which accelerates OBs differentiation. It may also interfere with extracellular signal-regulated kinase (ERK)/MAPK and NF-κB signaling to block RANKL-induced OCs differentiation [101]. Zoledronic acid inhibits FBXO9 (F-box protein 9) expression and increases OCs ferroptosis by triggering FBXO9-mediated ubiquitination and P53 degradation, thereby decreasing p53 protein stability [102].

## Ferroptosis in T2DM

The relationship between DM and ferroptosis is significant. T2DM is characterized by impaired pancreatic β-cell function and peripheral insulin resistance [103]. T2DM is characterized by the progressive loss of insulin secretion in the context of insulin resistance. Studies have shown that individuals with DM often exhibit imbalances in iron metabolism and increased serum circulating iron and FTN levels compared with healthy individuals [104–105]. Iron overload is considered a contributing factor to the worsening of T2DM [106].

### Adipocyte metabolism

This study showed that obesity-associated T2DM entails insulin resistance and loss of β-cell mass. Adipocyte mitochondrial dysfunction increases cell mass during obesity-related insulin resistance. And mitochondrial dysfunction in the adipose tissue is a key determinant of the etiology of type 2 diabetes [107]. Iron, an important regulator of energy metabolism, is primarily found in adipose tissue. Iron deficiency and iron overload are considered important causes of chronic metabolic diseases (such as T2DM or obesity) [108] Studies have shown that mice fed with rich iron diets showed upregulation of IR-related adipokines because an iron-enriched diet induces insulin resistance and hypertriglyceridemia and affects visceral adipose tissue metabolism by a mechanism involving hepcidin up-regulation [109]. FTMT is a mitochondrial matrix protein that chelates iron. Induction of FtMT in adipocytes promotes local ROS damage during obesity. It activates the mitochondrial stress response in adipocytes [108]. This response may induce ferroptosis during adipocyte metabolism, aggravating IR. In obesity, abnormal adipocyte metabolism is related to IR owing to adipocyte mitochondrial dysfunction. However, the association between adipocyte metabolism, ferroptosis, and DOP remains unclear. Obesity may be the most relevant factor affecting adipocyte metabolism.

### Liver metabolism

The relationship between iron metabolism, T2DM, and liver disease is complex. Iron main store in hepatocytes which play a dual role in iron metabolism [110]. In addition, hepatocytes secrete an iron regulatory hormone (hepcidin). Insufficient hepcidin expression results in iron overload, which triggers ROS synthesis which may have a relationship between ferroptosis pathogenesis of β cell exhaustion and IR-mediated T2DM [17, 111]. The end-stage liver disease would lead to the massive liver iron overload when the hepcidin-producing liver mass are losing [112]. Meanwhile iron overload may affect the development of liver fibrosis by aggravating lipid peroxidation [113]. Ferroptosis is involved in hepatic fibrosis and leads to liver injury in db/db mice, which may be attributed to increased ROS and lipid peroxidation, as well as iron deposition. Suppression of ferroptosis in hepatocytes by the Nrf2/HO-1 pathway. Moreover, liraglutide, widely used to treat obesity and T2DM, can prevent the occurrence of oxidative stress, iron overload and ferroptosis by activating Nrf2/HO-1, thereby improving liver fibrosis [114–115]. Recent studies have revealed an increased level of ferroptosis in hepatic cells during the progression of nonalcoholic fatty liver disease (NAFLD). NAFLD is closely associated with insulin resistance T2DM. And metformin can modulate the xCT/GPX4/ACSL4 axis to alleviate hepatic cell ferroptosis in T2DM-related NAFLD [116]. But the connect of ferroptosis, DOP and liver disease was still to find out. Maybe serious liver disease lead massive liver iron overload that cause ferroptosis of β cell exhaustion and IR-mediated T2DM which would affect DOP [117].

### Ferroptosis in β-cells

Ferroptosis is associated with insulin secretion dysfunction in pancreatic β-cells. The function of pancreatic islets can be impaired by proferroptotic factors even before β-cells die [118]. Iron plays a crucial role in pancreatic β-cells insulin secretion. Iron intake is mainly derived from the diet [119]. Iron is taken up by the cell’s divalent metal transporter 1 (DMT-1) in the form of free Fe^2+^ and the transferrin receptor (TFR) is the main carrier of iron into the cell, binding to iron to form a trivalent iron complex (Tf-Fe^3+^), which is then transported into tissues [28], and Tf-Fe^3+^ is taken up by pancreatic β-cells via DMT-1 [120]. Fe^2+^ is retained in the labile iron pool (LIP) where it is sequestered by ferritin, a unique cytoplasmic iron storage protein [121]. Fe^2+^ bound to FTN to synthesize iron-dependent proteins in the cytoplasm or mitochondria [122]. Extracellularly, pancreatic β-cells release hepcidin, which induces the internalization of binding transferrin [123–124]. Studies have shown that hepcidin improves pancreatic β-cells function by alleviating mitochondrial iron overload [125]. Tf mediates a positive feedback mechanism for iron regulation during glucose-stimulated insulin secretion [126]. Therefore, pancreatic β-cells play an important role in controlling iron homeostasis by avoiding excessive free Fe^2+^.

Ferroptosis is the process of non-apoptotic cell death due to excessive iron uptake associated with a reduction in mitochondrial volume, and is characterized by iron accumulation, lipid peroxidation, and reduced GPX4 expression [23]. For example, the pathogenesis of hereditary hemochromatosis (HH) involves iron deposition in pancreatic β-cells, leading to pancreatic β-cell death and consequent high glucose (HG) conditon, even DM [127]. As pancreatic β-cells express low levels of antioxidant enzymes such as SOD, GSH peroxidase, and catalase [128], pancreatic β-cells are susceptible to oxidative stress, have relatively low antioxidant capacity, and are prone to ferroptosis. Studies have shown that ferroptosis can be induced in rat’s pancreatic β-cells by silencing GPX4 and exposure to tert-butyl hydroperoxide (tert-BHP). It means GPX4 or Fer-1 effectively attenuates tert-BHP-induced pancreatic β-cell death, and thus, GPX4 is essential for pancreatic β-cell survival [129]. Erastin and RSL3 induce pancreatic β-cell ferroptosis, leading to impaired islet function, whereas Fer-1 restores impaired glucose-stimulated insulin secretion (GSIS) [130]. Cryptochlorohydric acid (CCA) exerts potent antidiabetic effects by activating the cystine/Xc-system/GPX4/Nrf2 pathway and inhibiting NCOA4 to suppress ferroptosis [131]. Ferroptosis is involved in pancreatic injury, glucose tolerance, iron deposition, and diabetic symptoms of T2DM in mice, whereas inhibition of ferroptosis can restore islet function [132]. Thus, the pathogenesis of T2DM is closely linked to pancreatic β-cell ferroptosis.

**Fig. 3:**
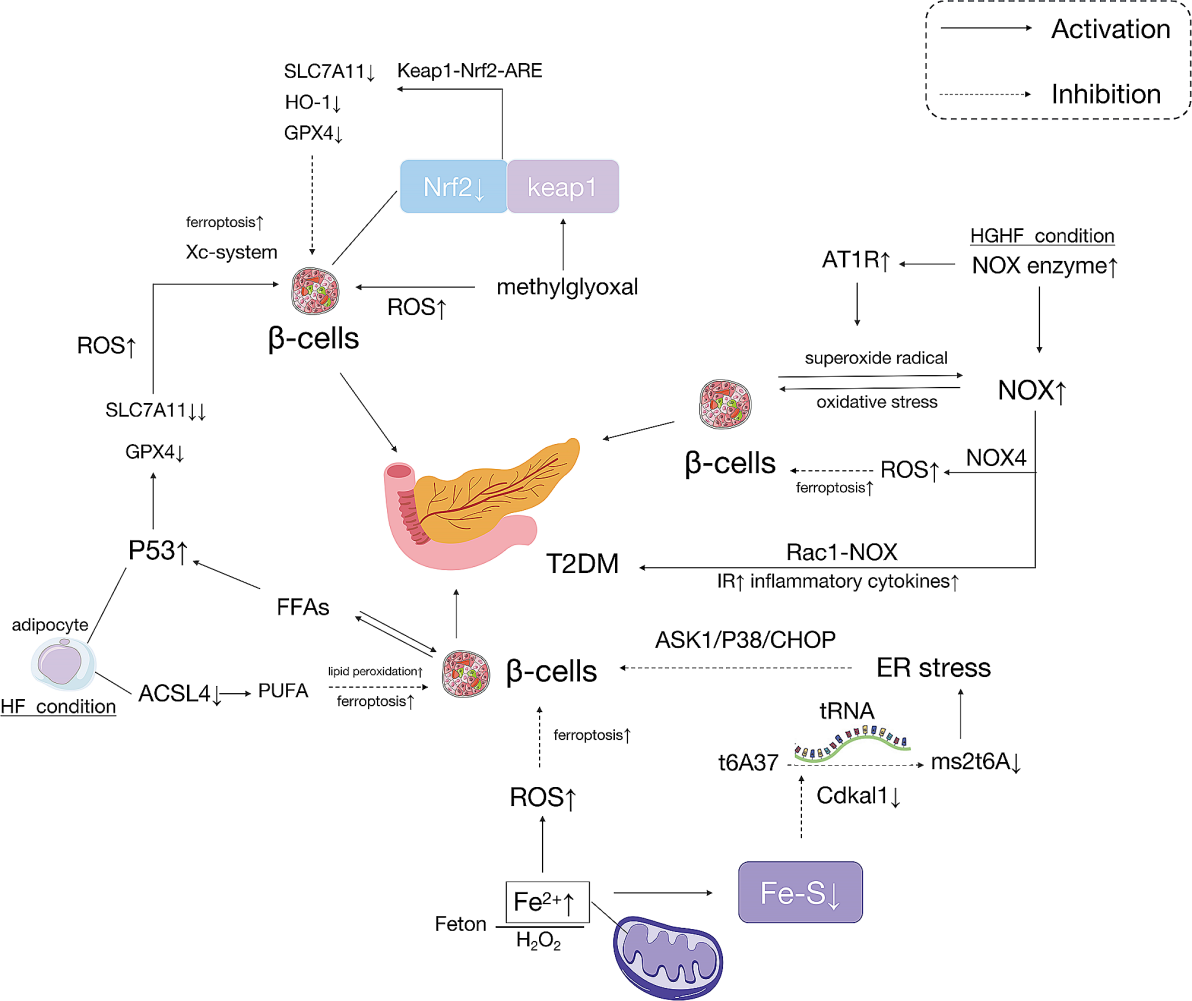
Ferroptosis occurs in pancreatic β-cells

The mechanisms by which ferroptosis occurs in pancreatic β-cells in the context of T2DM may involve several pathways (shown in Fig. 3):

(1) A deficiency of iron-sulfur clusters synthesis reduces the function of Cdk5-regulatory subunit-associated protein 1-like 1 (Cdkal1). Cdkal1 catalyzes the methylthiolation of N6-threonylcarbamoyl adenosine 37 (t6A37) in cytosolic tRNALysUUU to generate 2-methylthio-N6-threonylcarbamoyl adenosine (ms2t6A37), which is required for accurate translation of lysine codons in proinsulin. Proinsulin is misread because of a deficiency in the synthesis of Fe-S, leading to a decrease in insulin content and secretion, as well as transport to the endoplasmic reticulum, causing endoplasmic reticulum stress [12]. This can trigger endoplasmic reticulum (ER) stress. ER stress can lead to apoptosis of pancreatic β-cells through the activation of apoptosis signal-regulating kinase 1 (ASK1) and the ASK1/P38/CCAAT-enhancer-binding protein homologous protein (CHOP) signaling pathway [133–134]. This contributed to the development of T2DM [12]. In addition, Fe^2+^ accumulation in the mitochondria leads to a deficiency of the Fe-S clusters, resulting in increased ROS levels in the mitochondria, which may subsequently lead to pancreatic β-cells ferroptosis due to the accumulation of lipid peroxides [12].

(2) ACSL4 exists in human and rat pancreatic β-cells and plays a role in the modification of fatty acids (FAs) within insulin secretion granules [135]. In studies involving mice fed a high-fat diet, specific inhibition of ACSL4 led to protection against insulin resistance [136]. Thus, ACSL4 could be an important lipid-metabolizing enzyme associated with pancreatic β-cells ferroptosis, potentially mediating ferroptosis through the generation of lipid peroxidation via PUFAs.

(3) P53 is a tumor suppressor gene that induces cell cycle arrest, apoptosis, and senescence [137]. When β-cells are treated with free fatty acids (FFAs), p53 is activated ROS production is increased [138], and upregulation of p53 in adipose tissue leads to an inflammatory response that results in insulin resistance [139]. The underlying mechanism may be that the activation of p53 severely reduces the protein level of SLC7A11. P53 is closely related to GPX4, the only member of the GPX family that can resist peroxide damage. GPX4 mediates oxidative stress and regulates ferroptosis [140]. P53 severe activation will reduce the SLC7A11 protein levels, which can lead to GPX4 deficiency and increased ROS production in pancreatic β-cells, which may mediate ferroptosis in the Xc-system of pancreatic β-cells, triggering steatosis causing T2DM.

(4) NAF-1 is a highly conserved [2Fe-2 S] protein of the NEET protein family localized to mitochondria, that is, the ER and mitochondria-associated membrane junctions. In a rat islet tumor clonal cell model (INS-1) lacking NFA-1, insulin secretion was impaired in the presence of increased lipid peroxidation, GPX4 expression, and mitochondrial death characteristic of ferroptosis. In contrast, combined treatment with ferrostatin-1 (iron chelator deferiprone) and the glutathione precursor N-acetylcysteine promoted mitochondrial and ER structural repair, and reduced mitochondrial destabilizing iron and ROS levels. It also improves the growth of NFA-1-deficient INS-1 cells and restore glucose-stimulated insulin secretion [141]. While the genes encoding NAF-1 are WFS-1 and mutant CISD2, this gene is present in Wolfram syndrome type 2 (WFS-T2), so the pancreatic β-cells ferroptosis due to NAF-1 deficiency may be the mechanism of diabetes associated with WFS-T2.

(5) In response to ROS, the cysteine residue of Keap1 is modified to dissociate between Nrf2 and Keap1 or to activate Nrf2 by activating its phosphorylation by a variety of kinases, thus enhancing the stability of Nrf2 and translocating it to the nucleus. It attaches to the promoter region and activates downstream molecules, including NAD(P)H: quinone oxidoreductase 1 (NQO1), HO-1, GPX4, SLC7A11 [142]. Among the downstream target molecules of Nrf2, GPX4 and SLC7A11 are closely associated with ferroptosis. Therefore, ferroptosis may occur when the antioxidant system collapses if excess ROS leads to peroxidation, which exceeds the antioxidant capacity of the Nrf2 pathway. Nrf2 protects β-cells from metabolic stress-induced oxidative damage. Because Nrf2 is a central regulator of β-cell mass by managing the level of cellular redox. One study found that the contribution of Nrf2 activity to the construction of β-cell mass included the maintenance of insulin levels [143]. When β-cells lack Nrf2 activity, insulin immunostaining and insulin content decrease. And Nrf stimulated the proliferation of the rat INS-1 and primary pancreatic β-cells [144]. In addition, Bollong [145] et al. found that methylglyoxal, a metabolite of glycolysis, covalently modified the Keap1 cysteine C151, leading to dimerization and Nrf2 activation [146]. Methylglyoxal itself can also generate ROS in β-cells, and increased glycolysis activates Nrf2. Thus, the Keap1-Nrf2-ARE signaling pathway is closely related to pancreatic β-cell ferroptosis. Nrf2 activity deficiency, along with diminished GPX4 and SLC7A11 levels among downstream target molecules, lowers the antioxidant capability within pancreatic β-cells. This reduction heightens pancreatic β-cells ferroptosis, contributing to diminished insulin mass or content, which will lead to a decrease in insulin mass or content decrease, which may induce or aggravate T2DM.

(6) The NADPH oxidase (NOX) protein is a membrane-associated multi-subunit enzyme that transfers electrons across biological membranes and is required for superoxide production. Evidence suggests that NOX is a major source of mitochondrial ROS in β-cells. In addition, NOX1, NOX2, NOX4, and a number of other cytoplasmic regulators and their homologs NOXO1 and NOXA1 are expressed in pancreatic β-cells [147–149]. Superoxide radicals produced by NOXs can contribute to pancreatic β-cell damage and induce intracellular oxidative stress [150–151]. When cells respond to chronic hyperglycemia, the expression of NOX enzymes is upregulated, activating angiotensin II type 1 receptor (AT1R) and increasing superoxide production [152–153]. Hyperglycaemia and AT1R-induced pro-inflammatory cytokines in human pancreatic islets lead to impaired insulin secretion and inflammation [154–155]. AT1R inhibition selectively downregulates NOX, which in turn inhibits oxidative stress, improving β-cell insulin secretion and reducing β-cell apoptosis [156]. In addition, exposure of β-cells to HG induces an increase in CD36 expression on the plasma membrane, leading to enhanced uptake of FFAs and a robustly upregulated NOX activity mediated by RAC1 induction.

Simultaneously, elevated FFAs’ levels lead to ceramide accumulation and ceramide-induced defects in β-cell function and promote insulin resistance, obesity, and inflammatory cytokines through the involvement of the RAC1-NOX pathway [157–158]. NOX4 contains a catalytic subunit that transfers electrons from NADPH to oxygen, resulting in ROS formation [159]. NOX4 has been identified as a mitochondrial targeting sequence and has been shown to be a major source of ROS in various cell types [160]. Ferroptosis is closely associated with ROS, mitochondria, and ROS-generating enzymes such as NOX4, which are known sources of ROS in bone tissue [161–162]. Recent studies have shown that both mitochondria and NOX4 are involved in ferroptosis [163–164]. NOX4 generates ROS using NADPH as a substrate, and one study showed that the activation of NOX4 leads to the overproduction of lipid peroxides and induces ferroptosis in glioma cells [164]. However, whether NOX4 or other NADPH oxidases cause ferroptosis in β-cells and thus induce or exacerbate diabetes needs further validation.

## Ferroptosis in T2DOP

OP can be classified as primary, secondary, or idiopathic. DOP are among the most common secondary OPs [5], and are closely associated with diabetes. Research recommends that blood 25-hydroxyvitamin D (25-OH-VD) levels should be tested in clinically admitted (living in nursing homes or residential care) patients with diabetes at risk of falls and fractures. This helps to rapidly identify treatable causes of falls and fractures. Studies have also found a strong correlation between poor glycemic control and increased bone fragility. The HbA1c threshold was > 9% (75 mmol/mol) for patients with T2DM and > 9% (63 mmol/mol) for patients with T1DM [165]. In addition, research suggests that DM leads to the accumulation of advanced glycation end products (AGEs) in various organs, including bones. In terms of bone structural properties, the accumulation of AGEs in the bone and the crosslinking of collagen fibers (intra- and inter-crosslinks) reduce bone biomechanical properties by increasing material stiffness [166]. Studies in Zucker diabetic Sprague-Dawley (ZDSD) rats, an animal model of T2DM, suggested that although mineralization remains normal, bone strength decreases significantly with the duration of diabetes [167]. HG conditions in T2DM severely impair the biological function of OBs, leading to increased mitochondrial double-layer density, a reduced number of mitochondrial cristae, and accumulation of ROS, and lipid peroxidation. This results in OBs exhibiting oxidative stress and lipid peroxidation, which accelerates apoptosis and autophagy [168]. Lin et al. found significantly elevated serum ferritin levels in a rat model of diabetic OP. SLC7A11 and GPX4 expression is significantly reduced [87]. These findings suggest that the HG environment in T2DM may induce ferroptosis in OBs, disrupting bone homeostasis and leading to OP. Therefore, bone cell ferroptosis may play an important role in T2DOP. This article reviews the relationship between T2DM and OP, and discusses the potential mechanisms underlying ferroptosis in T2DOP.

### OP’s ferroptosis in HG environment

#### OBs’ ferroptosis in HG environment

HG toxicity may directly affect the differentiation and function of OBs and OCs, leading to impaired bone remodeling and OP development. Studies have observed that the differentiation of BMSCs into OBs is inhibited in the serum of patients with T2DM [169]. High blood glucose levels lead to reduced OB capacity and decreased bone mineralization. Research on newly diagnosed patients with T2DM suggests that the HG microenvironment may lead to impaired OB maturation and bone formation [170]. Similarly, iron overload downregulates the expression of RUNX2 and its targets, alkaline phosphatase and osteocalcin, in BMSCs and OBs progenitors, thereby inhibiting OBs formation [79]. HG conditions may also affect iron metabolism in OBs. Excess iron reduced the viability of MC3T3 cells and induced apoptosis. Excessive iron may partially inhibit OB activity and disrupt OB differentiation and mineralization [85]. HG induces ferroptosis in OBs by activating the METTL3/ASK1-p38 signaling pathway [171]. Therefore, abnormal iron metabolism under HG conditions may affect OB differentiation, leading to ferroptosis. There is evidence of ferroptosis in OBs in HG environments. HG/high oxygen conditions and the NFκB pathway promote the expression of NOX4. The expression of NOX4 is negatively regulated by several antioxidant factors [171]. Elevated levels of NOX4 result in the production of ROS and accumulation of lipid peroxidation, leading to morphological and functional impairments in OBs mitochondria and promoting ferroptosis in OBs [21]. Activating Transcription Factor 3 (ATF3), a member of the ATF/Cyclic AMP Response Element-Binding protein (CREB) transcription factor family, plays a crucial role in transcriptional repression and promotion with a dual function of regulating cell apoptosis, proliferation, and the cell cycle. HG conditions induce the upregulation of ATF3, leading to the reduced expression of SLC7A11 and lower intracellular levels of GSH and extracellular glutamate. Therefore, ferroptosis in OBs under HG conditions is mediated by the inhibition of Xc-system activity by ATF3 [172]. Under HG conditions, FtMt overexpression reduced ferroptosis in OBs, whereas FtMt inhibition induced mitochondrial autophagy through the ROS/PINK1/Parkin pathway, resulting in increased ferroptosis [63]. Additionally, amino acids and reducing sugars undergo non-enzymatic reactions in the physiological environment to form AGEs. Prolonged high blood glucose levels lead to the significant accumulation of late-stage AGEs in the body [166]. Research suggests that AGEs promote ferroptosis in OBs, thereby contributing to OP development [173].

#### OCs’ ferroptosis in HG environment

Several studies have indicated that HG mircoenvironment stimulates the differentiation of OCs, thereby increasing their capacity for bone resorption [174]. Additionally, clinical research has observed elevated levels of tartrate-resistant acid phosphatase in the bloodstream of patients with T2DM, suggesting increased OC activity [175]. In animal models, studies have revealed elevated bone resorption in T2DM rats compared to normal glucose control groups. This indicates higher OC activity in T2DM models [176–177]. Under HG conditions, factors including TNF-α, macrophage colony-stimulating factor, receptor activator of RANKL, and vascular endothelial growth factor-A are heightened in diabetic mice. These factors facilitate the differentiation and activation of OCs [178–179]. Studies have indicated that the differentiation of bone marrow monocytes into OCs is stimulated by the RANKL, which enhances NOX4 mRNA expression and ROS production. In NOX4-deficient mice, RANKL was ineffective in stimulating the expression of two recognized transcription factors for osteoclast differentiation, namely the nuclear factor of activated T cells cytoplasmic 1 (NFATC1) and activator protein 1; resulting in augmented bone mass and thicker trabecular bone structure in NOX4-deficient mice [180–181]. The hyperglycaemic/hyperoxic environment and the NF-κB pathway promote the expression of NOX4, which is negatively regulated by various antioxidant factors [160, 171]. Under HG conditions, the NFκB pathway activation leads to increased NOX4 expression, which may in turn activate OCs differentiation transcription factors via RANKL, thereby increasing OCs differentiation. The build-up of AGEs in the body during the later stages of T2DM stimulates OCs activity, resulting in a higher bone resorption area, and thus increased bone resorption [182]. The presence of AGEs causes oxidative stress responses and triggers the activation of the receptor for AGEs (RAGE), leading to RANKL-mediated OCs formation [183–184]. The NFκB also plays a role in oxidative stress and increased OCs activity, while simultaneously decreasing OBs differentiation [185]. As blood glucose levels increase, the levels of additional proteins including AGEs, pro-inflammatory cytokines, and ROS also increase [186]. ROS activation triggers the intracellular MAPK signaling pathway. Activation of ROS/MAPKs/NF-κB/NLRP3 causes OC-mediated bone loss in patients with DOP [174].

Furthermore, in vitro studies using RAW264.7 cells indicate that HG reduces OCs autophagy, thus amplifying OCs formation [187]. Ferritin is responsible for storing excess cellular iron. When cells are iron deficient, they may degraded, a phenomenon commonly referred as “ferritinophagy”. This leads to increased cell sensitivity to ferroptosis caused by Fe^2+^ in cells [188]. Owing to their high energy demands to function as acid-secreting and absorptive bone cells, OCs are abundant in mitochondria [189]. Therefore, fully developed OCs require higher amount of free intracellular iron than other bone cells. The iron-chelating agent deferoxamine mesylate (DFO) impedes OC formation in vitro [94]. Ni et al. discovered that ferroptosis impacts OCs during the differentiation phase when stimulated by RANKL and overexpressed TfR1, causing a reduction in the iron concentration prompted by the decreased activity of urocanase [190]. Owing to OCs’ iron-dependent nature of OCs, which is similar to that of cancer cells, they are more prone to iron-triggered ferroptosis. Research findings indicate that the artemisinin compound ART enhances the generation of MDA and 4-HNE, resulting in the manifestation of mitochondrial ferroptosis traits and prompting ferroptosis in OCs, which hinders their differentiation [191]. OCs differentiation increases under high glucose conditions, thereby enhancing the bone resorption capacity. Furthermore, it is possible that the OCs in DOP undergo ferroptosis, presenting a promising therapeutic avenue for DOP through the induction of OC death and inhibition of their differentiation.

### Insulin resistance and deficiency in OP-related ferroptosis

Razny et al. discovered that insulin resistance results in reduced mRNA expression in OBs generation, increased mRNA expression of OBs ogenesis inhibitors, and suppression of miRNAs, such as miRNA-29b and miRNA-181a. These factors are responsible for promoting OBs generation and osteogenic differentiation of stem cells. Consequently, defects occur in the OBs of patients [192]. Insulin-like growth factor 1 (IGF-1) secreted by pancreatic β-cells plays a crucial role in bone mass and is strongly associated with vertebral fractures. Impaired IGF-1 function, reduced synthesis of active vitamin D, inhibition of bone calcitonin synthesis and secretion, and increased ceramide synthesis can adversely affect the bone microstructure and material properties. Subsequently, bone strength is reduced, and the likelihood of fracture is elevated [193]. As T2DM advances and insulin levels decrease, metabolic disruptions occur in glucose, fat, and protein, resulting in a negative nitrogen balance in the body. This allows the compensatory breakdown of bone collagen and reduces its synthesis, ultimately resulting in decreased bone formation. Additionally, insulin restricts cyclic adenosine monophosphate (cAMP). cAMP motivates bone resorption and reduces bone calcium salt deposition. Insulin deficiency diminishes this inhibitory effect, boosts cAMP activity, and consequently enhances bone resorption, culminating in OP [194]. Osteocalcin is a non-collagenous protein that is mainly synthesized and secreted by OBs. Its primary role is to uphold the rate of bone mineralization, foster bone metabolism, and indicate the level of OBs’ activity [195]. Insulin deficiency may impede OBs from synthesizing osteocalcin, which may decelerate bone mineralization, metabolism, and activity, and reduce bone formation.

In addition, insulin directly facilitates the reabsorption of calcium, phosphate, and magnesium in the renal tubules. When insulin is inadequate, renal tubules reabsorb fewer minerals, resulting in their loss, decreased concentration of calcium in the blood, and the release of calcium from the bones. This leads to reduced bone density and the onset of OP [196]. Research indicates that increased ATF3 expression leads to cell damage, including apoptosis of pancreatic β-cells, the generation of ROS, and high levels of glucose or FA associated with complications in diabetes. High levels of ATF3 are linked to stress-induced apoptosis of pancreatic β-cells [197]. Under HG conditions, ATF3 upregulation inhibits the activity of the Xc-system and causes ferroptosis in OBs [172]. Therefore, ATF3-mediated ferroptosis of pancreatic β-cells occurring with insulin deficiency may be linked to ferroptosis in OBs associated with OP.

### OP’s ferroptosis in diabetic microvascular disease

Microvascular diseases in DM are characterized by augmented capillary permeability, thickening of microvascular basement membranes, destruction of endothelial cells, and platelet adhesion and aggregation, culminating in the creation of microthrombi or microvascular occlusion. This can have an impact on multiple organs in the body and diseases, including the diabetic nephropathy(DN), diabetic retinopathy (DR), and diabetic peripheral neuropathy (DPN) [198]. Diabetes complications, such as damage to vascular dilation, vascular calcification and vascular regeneration, can affect the development of OBs progenitor cells within the hematopoietic niche, as well as the transportation of OBs (or periosteal cells) and OCs to the Haversian canals [199]. Impaired blood flow and damage to neovascularization may lead to decreased OBs formation and reduced bone remodeling activity, ultimately resulting in decreased bone mass and delayed fracture healing. Furthermore, numerous studies have indicate that the AGE/RAGE signaling pathway has a significant impact on vascular calcification. In peripheral vascular smooth muscle cells (VSMCs), AGEs activate RAGE that leads to a sequence of signaling pathways involving MAPK, TGF-β, and NF-κB. This leads to the downregulation of VSMC markers and activation of an OBs program, resulting in increased expression of Runx2 and osteocalcin, as well as enhanced alkaline phosphatase activity, ultimately leading to VSMC calcification [200]. Iron overload induces ROS accumulation, which activates intracellular MAPK signaling pathways. ROS/MAPKs/NF-κB/NLRP3 activation results in OCs-mediated bone loss in DOP [174]. Bone vascular calcification is linked to the ROS/MAPKs pathway, which can be activated by iron overload-induced ROS accumulation, affecting OBs funtion or activity. Additionally, AGEs contribute to vascular calcification and also induce ferroptosis in OBs [201]. These findings suggest a potential association between microvascular complications in diabetes and the ferroptosis of DOP.

## Summary

**Fig. 4:**
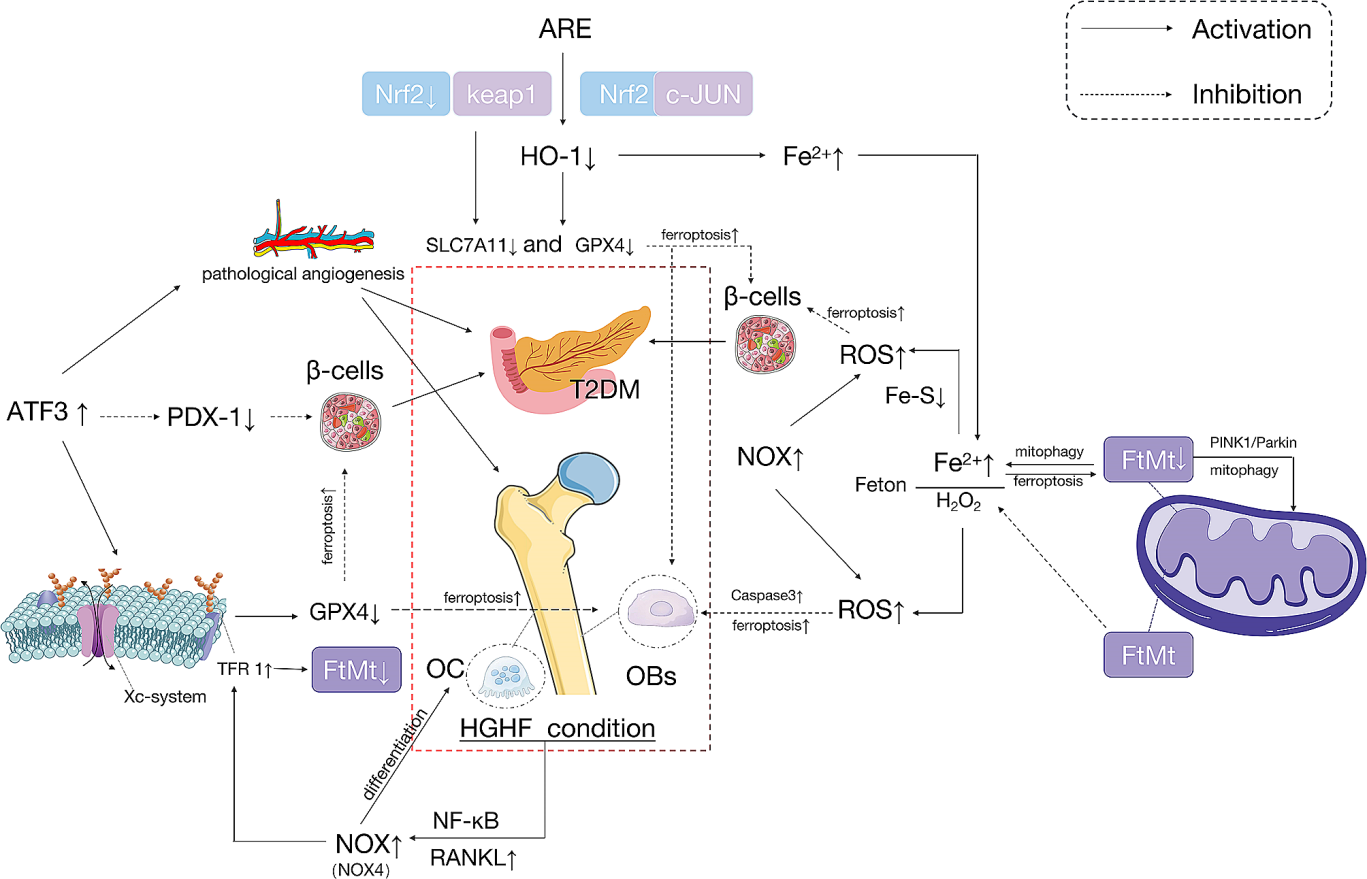
Ferroptosis in DOP

T2DM has a close relationship with the development of OP. Changes in the HG microenvironment, insulin resistance, insulin insufficiency, and microangiopathy can exacerbate the progression of OP in patients with T2DM. The HG environment in T2DM may activate bone cells ferroptosis, disturbing bone homeostasis and leading to OP. Furthermore, cell ferroptosis could impact both pancreatic β-cells and bone cells concurrently through numerous pathways, significantly impacting the advancement of T2DM and OP. However, the mechanism underlying ferroptosis in DOP remain unclear. We examined the possible mechanisms of ferroptosis in DOP (shown in Fig. 4).

(1) ATF3/Xc-system/GPX4.

Jang’s research found that ATF3, by binding to ATF3 response elements in its promoter, suppresses the expression of pancreatic duodenal homeobox 1 (PDX-1) in pancreatic β-cells, thereby inhibiting pancreatic β-cells function [202]. These findings suggest that ATF3 play an important role in the onset and development of T2DM. Okamoto et al. reported an increase in ATF3 expression in renal glomeruli and aortic endothelial cells of a DN, rat model, suggesting that ROS-related vascular complications in diabetes may involve ATF-mediated pathological angiogenesis [203]. ATF3 also mediates the Xc-system/GPX4 pathway in OB ferroptosis under HG conditions. Therefore, T2DM may affect DOP progression via the ATF3-mediated signaling pathway.

(2) NRF2/HO-1/GPX4/SLC7A11.

The T2DM microenvironment significantly promotes ferroptosis in bone cells in vitro with increased lipid peroxidation and iron overload. According to RNA sequencing results, HO-1 expression was significantly upregulated in bone cells. HO-1 is critical for DOP ferroptosis [13]. Yang et al. found that there is more lipid peroxidation in vivo in a DOP rat model, suggesting that the HG microenvironment can induce ferroptosis in bone cells. They believe that in the DM microenvironment, HO-1 transcription is activated upstream by NRF2 and c-JUN, leading to the activation of HO-1, which catalyzes haem oxidation and generates a large amount of free iron [13]. In addition, research by Ma supported the theory that HO-1 mediates HG-induced ferroptosis in bone cells [81]. HO-1 activation and ferroptosis are mutually dependent and may lead to an endless cycle of mutual promotion [204]. Targeting ferroptosis (injection of the ferroptosis inhibitor, Fer-1) or HO-1 (injection of zinc protoporphyrin, an HO-1 inhibitor) may reverse ferroptosis in bone cells during DOP by interfering with lipid peroxidation, ultimately reversing bone loss. Among the downstream targets of NRF2, GPX4 and SLC7A11 are important markers of iron deficiency anemia. The importance of NRF2 transcriptional targets in the regulation of lipid peroxidation, inhibition of free iron accumulation, and glutathione synthesis and metabolism, which are all related to ferroptosis, is becoming increasingly clear [33]. MaR1 treatment alters the NRF2 pathway for SLC7A11/GPX4 signaling, protecting OBs from the effects of ferroptosis in T2DM, thereby improving their bone-forming capacity [205]. In T2DOP, HG induces ferroptosis by increasing ROS/lipid peroxidation/GSH depletion. Melatonin significantly reduces iron-deficiency anaemia in vitro and in vivo and increases the osteogenic capacity of MC3T3-E1 cells by activating the Nrf2/HO-1 pathway [81]. Studies have suggested that pancreatic iron overload is involved in the pathogenesis of T2DM. And vitamin D may inhibit ferroptosis in pancreatic β-cells through the NFκB-DMT1 signaling pathway [205]. In addition, the VDR agonist (1,25(OH)2D3) can inhibit ferroptosis in bone cells by activating the Nrf2/GPX4 signaling pathway [90]. Therefore, vitamin D drugs may protect against DOP development.

(3) NOX4/NF-κB/RANKL.

NOX is a major source of mitochondrial ROS in β-cells. The superoxide radicals produced by NOX can damage pancreatic β-cells and induce intracellular oxidative stress [150–151]. When cells are exposed to chronic hyperglycemia, NOX expression is upregulated. The NOX downregulation can suppress oxidative stress, thereby improving pancreatic β-cells insulin secretion and reducing β-cells apoptosis [160]. Increased NOX4 expression promotes ferroptosis in OBs [21]. In an HG environment, the NFκB pathway promotes NOX4 expression, activates RANKL, induces excessive TfR1 expression, and significantly reduces iron levels, which can lead to ferroptosis in OBs and promote OCs differentiation. Whether NOX4 or other NADPH oxidases cause ferroptosis in pancreatic β-cells, thereby inducing or exacerbating diabetes, requires further investigation. However, the HG microenvironment of T2DM may increase NOX and affect OP through the NOX4/NF-κB/RANKL pathway.

(4) Fenton reaction and iron autophagy.

Fe2 + interacts with H_2_O_2_ through the Fenton reaction, causing lipid peroxidation and inducing ferroptosis in cells [206]. The Fenton reaction generates a large amount of hydroxyl radicals and other ROS, which stimulate the release of cytochrome C from the mitochondria and activate the key apoptosis protein Caspase3, promoting apoptosis of OBs [88–89]. Pancreatic β-cells are susceptible to oxidative stress and have a relatively low antioxidant capacity, making them susceptible to ferroptosis. The accumulation of Fe^2+^ in mitochondria leads to a lack of Fe-S clusters, resulting in increased mitochondrial ROS levels. The subsequent accumulation of lipid peroxidation products can lead to pancreatic β-cells ferroptosis.

Iron autophagy maintains intracellular iron balance under physiological conditions, but excessive activation of ferritinophagy leads to intracellular iron overload. Iron stored in ferritin can also be released into the cytoplasm, which is considered the starting point and ultimately triggers ferroptosis [27]. Studies have suggested that excessive autophagy and lysosomal activity promote ferroptosis through iron accumulation or lipid peroxidation [207]. FtMt suppresses ferroptosis in OBs by reducing the oxidative stress caused by excess iron ions [62]. In addition, OBs ferroptosis is promoted under FtMt deficiency condition. FtMt deficiency activates the ROS/PINK1/Parkin pathway, induces mitochondrial autophagy, and releases free Fe^2+^ into the cytoplasm, aggravating the Fenton reaction [206]. Therefore, a relationship between the Fenton reaction and iron autophagy have been implicated in OBs ferroptosis.

## Therapeutic opportunities

Although the mechanisms of T2DOP ferroptosis have been discussed above, the therapeutic opportunities of T2DOP target these mechanisms to promote cell recovery during ferroptosis. Ferrostatin-1 (Fer-1), a small-molecule drug, is an effective inhibitor of ferroptosis because of its ability to scavenge lipid peroxides. It can target ferroptosis or HO-1 to efficiently rescue osteocyte death in DOP by disrupting the vicious cycle between lipid peroxidation and HO-1 activation, eventually ameliorating trabecular deterioration [12]. Studies have shown that poliumoside (Pol) inhibits HGHF-induced bone degradation and ferroptosis by elevating GSH and decreasing malondialdehyde (MDA) levels, lipid peroxidation, and mitochondrial reactive oxygen species (ROS). Pol effective in treating T2DOP by activating the Nrf2/GPX4 signaling pathway to inhibit ferroptosis, which is beneficial for HGHF-treated BMSCs [208] Curcumin, a tetrahedral framework nucleic acid (tFNA) can also effectively inhibit ferroptosis by activating the NRF2/GPX4 pathway. It can enhance mitochondrial function by activating this pathway, induce the osteogenic differentiation of BMSCs in the diabetic microenvironment, as well as reduce trabecular loss and increase bone formation in a mouse model of DOP [209]. Maresin1 (MaR1), a derivative of one of the primary omega-3 fatty acids, docosahexaenoic acid (DHA), is an endogenous, pro-resolving lipid mediator produced mainly by macrophages. It was found that could enhance the expression levels of NRF2, GPX4 and SLC7A11 to cause the restraint of ferroptosis under hyperglycemic stimulation. MaR1 can activate the NRF2/GPX4 pathway in vivo and in vitro to alleviate high-glucose-induced ferroptosis greatly [82].

In addition, other compounds affect ferroptosis through different pathways. Vitamin K2 (VK2), a fat-soluble vitamin, can decrease the levels of mitochondrial ROS, lipid peroxidation, and malondialdehyde, and increase GSH in BMCS. It also restored bone mass and increased SIRT1 and GPX4 expression. Therefore VK2 may ameliorate T2DOP through the activation of the AMPK/SIRT1 signaling pathway to inhibit ferroptosis [210] An arabinomannan (PAAP-1B), a molecular weight of 14.0 kDa was isolated from Anemarrhena asphodeloides Bge, suppressed ferroptosis in advanced glycation end product-induced osteoblasts by decreasing MDA, mitochondrial ROS levels, and lipid peroxidation, while reversing the downregulation of SLC7A11 and GSH expression [211]. Eldecalcitol (ED-71), a novel active form of vitamin D, can attenuate the over expression of hypoxia-inducible factor 1α (HIF1α) induced by high glucose levels, also increase GPX4 levels. It may improve osteogenic disorders caused by diabetes such as DOP [212].

However, there are still many potential therapeutic opportunities to promote the ability of cells to recover from oxidative stress, which is beneficial for treating T2DOP. Ferroptosis-specific antagonists for the treatment of T2DOP and the lack of research are not listed in this paper. Meanwhile, herbs or their active ingredients (quercetin, curcumin, cryptochlorogenic acid, resveratrol, Platycodonopsis saponin D, Astragaloside IV) can also inhibit ferroptosis [213], which provides a new idea for T2DOP treatment.

In conclusion, T2DM is closely associated with OP development. Ferroptosis affects the progression through multiple pathways. Regulation of ferroptosis in pancreatic β-cells and bone cells may be a new target for the treatment of DOP.

### Electronic supplementary material

Below is the link to the electronic supplementary material.

Supplementary Material 1

Supplementary Material 2

Supplementary Material 3

Supplementary Material 4

Supplementary Material 5

## Data Availability

Due to its nature as a review article, all references are published articles. The data underlying this article are available in the Pubmed.

## Abbreviations

25-OH-VD: 25-hydroxyvitamin D
4-HNE: 4-Hydroxynonenal
AA: Arachidonic acid
ACSL4: Acyl-CoA synthetase long-chain family member 4
AdA: Adrenergic acid
AGEs: Advanced glycation end products
AREs: Antioxidant response elements
ARS: Artesunate
ASK1: Apoptosis signal-regulating kinase 1
AT1R: Angiotensin II type 1 receptor
ATF3: Activating Transcription Factor 3
ATF4: Activating transcription factor 4
BMSCs: Bone marrow mesenchymal stem cells
cAMP: Cyclic adenosine monophosphate
CCA: Cryptochlorohydric acid
Cdkal1: Cdk5-regulatory subunit-associated protein 1-like 1
CHOP: CCAAT-enhancer-binding protein homologous protein
CoQ10: Coenzyme Q10
CoQ10H2: Coenzyme Q10H2
CoQH2: Coenzyme QH2
CREB: Cyclic AMP Response Element-Binding protein
CYP450: Cytochrome P450
DFO: Deferoxamine mesylate
DHODH: Dihydroorotate dehydrogenase
DM: Diabetes mellitus
DMT-1: Divalent metal transporter 1
DN: Diabetic nephropathy
DOP: Diabetic osteoporosis
DPN: Diabetic peripheral neuropathy
DR: Diabetic retinopathy
ER: Endoplasmic reticulum
ERK: Extracellular signal-regulated kinase
FAC: Ammonium ferric citrate
FAs: Fatty acids
F-box protein 9: FBXO9
Fer-1: Ferrostatin-1
FFAs: Free fatty acids
FINO2: 1,2-dioxin cycloalkane
FPN: Ferroportin
FSP1: Ferroptosis inhibitory protein 1
FTH: Ferritin
FTH1: Ferritin heavy chain 1
FTL: Ferritin light chain
FtMt: Mitochondrial ferritin
GPX4: Glutathione peroxidase 4
GSH: Glutathione
GSIS: Glucose-stimulated insulin secretion
H2O2: Hydrogen peroxide
HG: High glucose
HGPA: High glucose and palmitic acid
HH: Hereditary hemochromatosis
HMOX1: Haem oxygenase 1
IGF-1: Insulin-like growth factor-1
IRE: Iron-responsive element
IREB2: Iron regulatory protein 2
IRP1: Iron regulatory protein 1
Keap1: Kelch-like ECH-associated protein 1
LIP: Labile iron pool
Lip-1: Liproxtatin-1
LOOH: Lipid peroxides
LOXs: Lipoxygenases
LPCAT3: Lysophosphatidylcholine acyltransferase 3
MAPK: Mitogen-activated protein kinase
M-CSF: Macrophage-colony stimulating factor
MDA: Malondialdehyde
METTL3: Methyltransferase-like 3
ms2t6A37: 2-methylthio-N6-threonylcarbamoyl adenosine
NAC: N-acetylcysteine
NADPH: Nicotinamide adenine dinucleotide phosphate
NCOA4: Nuclear receptor coactivator 4
NFATC1: Nuclear factor of activated T cells cytoplasmic 1
NLRP3: Pyrin structural domains of 3
NOX: NADPH oxidase
NOX4: NADPH oxidase 4
NQO1: NAD(P)H: quinone oxidoreductase 1
Nrf2: Nuclear factor E2-related factor 2
OB: Osteoblast
OBs: Osteoblasts
OC: Osteoclast
OCs: Osteoclasts
OP: Osteoporosis
OPG: Osteoprotegerin
PCD: Programmed cell death
PDX-1: Pancreatic duodenal homeobox 1
PE: Phosphatidylethanolamine
PL: Phosphatidylinositol
PUFA-COA: ACSL4 forms multiple unsaturated fatty acid coenzyme A
PUFA-PL: Phospholipid PUFAs
PUFAs: Polyunsaturated fatty acids
RAGE: Receptor for AGEs
RANKL: Nuclear factor κB ligand
ROS: Reactive oxygen species
RSL3: RAS-selective lethal small molecule 3
Runx2: Runt-related transcription factor 2
SAT1: Spermidine/spermine N1-acetyltransferase 1
SOD: Superoxide dismutase
T2DM: Type 2 diabetes mellitus
T2DOP: Type 2 Diabetic osteoporosis
t6A37: Cdkal1
TEM: Transmission electron microscopy
tert-BHP: Ert-butyl hydroperoxide
TFR: Transferrin receptor
VDR: Vitamin D receptor
VSMCs: Vascular smooth muscle cells
WFS-T2: Wolfram syndrome type 2
WHO: The World Health Organization
Xc system: Cystine/glutamate reverse transport system
ZDSD: Zucker diabetic Sprague-Dawley
