# Supplementary material for: Type 2 diabetic mellitus related osteoporosis: focusing on ferroptosis
Source: J Transl Med. 2024 Apr 30;22:409. doi: 10.1186/s12967-024-05191-x (PMC11064363; doi:10.1186/s12967-024-05191-x)
Supplement: Supplementary file 1 — Supplementary Material 1 [file 12967_2024_5191_MOESM1_ESM.pdf]

科研论文投稿

备案号	2023-3628	状态	审核通过
论文题目	The mechanism of ferroptosis in type 2 diabetic osteoporosis		
论文类别	综述	拟发表期刊	Free radical biology
拟投稿日期	2023-11-19		
是否有依托课题	否	是否使用大型设备	否
摘要	With the global population aging, type 2 diabetes mellitus (T2DM) and osteoporosis are becomin<br>increasingly prevalent. This brief review highlights the growing incidence of T2DM and osteoporo<br>s, particularly among the elderly, posing substantial challenges to public health and economies. Di<br>betic osteoporosis (DOP) is identified as a significant concern due to its association with fragility f<br>actures.The review explores the concept of iron death, a non-apoptotic cell death process depen<br>ent on intracellular iron, focusing on its role in T2DM. Iron-dependent lipid peroxidation, particula<br>y impacting pancreatic β-cells, contributes to diabetes. The intricate interplay between iron dysreg<br>lation, encompassing deficiency and overload, and osteoporosis is discussed, emphasizing how ex<br>essive iron accumulation triggers osteoblast iron death. In conclusion, this concise overview stress<br>s the need to understand the complex relationship between T2DM and osteoporosis, particularly<br>oncerning iron death. These insights into the pathogenesis of diabetic osteoporosis provide a foun<br>ation for future research and targeted interventions in this rapidly evolving field.		
是否涉及伦理	不涉及		

作者：

序号	署名顺序	姓名	年龄	通讯作者	第一作者	科室	署名单位	联系电话	职称
1	1	陈奕历			是	大德路总院老年骨折科病区	广东省中医院	13246807370	
2	2	赵稳			是		广州中医药大学		
3	2	胡安					广州中医药大学		
4	2	陈平				大德路总院老年骨折科病区		13580421617	副主任医师
5	3	杨冰				芳村医院正骨科病区	广东省中医院	18320727396	住院医师
6	4	范智荣				大德路总院老年骨折科病区	广东省中医院	13265354510	住院医师
7	5	祁冀				大德路总院老年骨折科病区	广东省中医院	15625173786	研究实习员

序号	署名顺序	姓名	年龄	通讯作者	第一作者	科室	署名单位	联系电话	职称
8	6	张文辉				大德路总院老年骨折科病区	广东省中医院	13650287638	
9	7	高欢欢				大德路总院老年骨折科病区	广东省中医院	15913177559	
10	8	喻秀兵				大德路总院老年骨折科病区	广东省中医院	13620404971	副主任中医师
11	10	陈路沅		是		其它单位			
12	11	王海洲		是		大德路总院老年骨折科病区	广东省中医院	13763332601	副主任中医师
13	12	陈海云				大骨科	广东省中医院		

附件列表：

名称	大小	文件操作
初稿docx	267kb	陈奕历 上传于 2023/11/12 23:31

流转记录

序	处理环节	处理人	意见	完成时间
1	开始			2023/11/12 23:34
2	申请	陈奕历		2023/11/12 23:34
3	论文作者确认	陈平	已确认	2023/11/21 12:38
3	论文作者确认	杨冰	已确认	2023/11/12 23:35
3	论文作者确认	范智荣	已确认	2023/11/12 23:43
3	论文作者确认	祁冀	已确认	2023/11/15 12:55
3	论文作者确认	张文辉	已确认	2023/11/13 01:07
3	论文作者确认	高欢欢	已确认	2023/11/13 07:53
3	论文作者确认	喻秀兵	已确认	2023/11/13 07:35
3	论文作者确认	王海洲	已确认	2023/11/15 10:19
3	论文作者确认	陈海云	已确认	2023/11/14 09:21
4	第一作者科室负责人审批	王海洲	同意	2023/11/21 12:40
5	科研处审批	曹倩	同意投稿	2023/11/22 10:48
6	结束			2023/11/22 10:48
