# Supplementary material for: Type 2 diabetic mellitus related osteoporosis: focusing on ferroptosis
Source: J Transl Med. 2024 Apr 30;22:409. doi: 10.1186/s12967-024-05191-x (PMC11064363; doi:10.1186/s12967-024-05191-x)
Supplement: Supplementary file 3 — Supplementary Material 3 [file 12967_2024_5191_MOESM3_ESM.pdf]

editage

Editing Certificate

This document certifies that the manuscript listed below has been edited to ensure language and grammar accuracy and is error free in these aspects. The edit was performed by professional editors at Editage, a brand of Cactus Communications. The author's core research ideas were not altered in any way during the editing process. The quality of the edit has been guaranteed, with the assumption that our suggested changes have been accepted and the text has not been further altered without the knowledge of our editors.

MANUSCRIPT TITLE

Type 2 diabetic Mellitus related Osteoporosis: focusing on ferroptosis

AUTHORS

Yili Chen; Wen Zhao; An Hu; Shi Lin; Ping Chen; Bing Yang; Ji Qi; Wenhui Zhang; Zhirong Fan; Xiubing Yu; Huanhuan Gao; Haiyun Chen; LuYuan Chen; Haizhou Wang

ISSUED ON

March 19, 2024

JOB CODE

QUXQG_6

THE EDITAGE Promise QUALITY GUARANTEE

Prabh Grewal
Senior Vice President - Editage

editage | helping you get published

Since 2002, Editage has helped over 430,000 authors publish around 1.2 million research papers in scholarly journals across over 1000 disciplines through editorial, translation, transcription, and publication support services. Editage is a brand of Cactus Communications (cactusglobal.com), a science communication and technology company.

CACTUS.

GLOBAL :
+1(833) 979-0061 | request@editage.com

CHINA :
400-120-3020或 021-6020-9400 | fabiao@editage.cn
