# Supplementary material for: Type 2 diabetic mellitus related osteoporosis: focusing on ferroptosis
Source: J Transl Med. 2024 Apr 30;22:409. doi: 10.1186/s12967-024-05191-x (PMC11064363; doi:10.1186/s12967-024-05191-x)
Supplement: Supplementary file 2 — Supplementary Material 2 [file 12967_2024_5191_MOESM2_ESM.docx]

Dear Prof.Francesco M. Marincola

RE: No.JTRM-D-24-00458

Thank you for your letter and for the Reviewers’ comments concerning our manuscript entitled, “Type 2 diabetic Mellitus related Osteoporosis: focusing on ferroptosis” by Yili Chen et al. We have carefully revised the manuscript text based on the reviewers’ suggestions and provided a point to point response to the comments. We here with submit the revised manuscript with changes highlighted and a “clean” copy for examination. We believe that the revisons based on the reviewers’ critique and manuscript edits have significantly improved the manuscript, and hope you find it acceptable for publication in Journal of Translational Medicine.

We are grateful to you for allowing us to revise our manuscript and look forward to hearing from you soon.

Sincerely

LuYuan Chen and Haizhou Wang

We thank the reviewers for their thorough examination of our manuscript, and the valuable and insightful comments provided. We have revised the manuscript accordingly and provided a point-to-point response to reviewers’ comments below.

our paper, as well as the important guiding significance to our further researches. We have studied these comments carefully and have made correction which we hope meet with approval. Revised portion have been marked using the highlight (with 'tracked changes'/coloured/underlines/highlighted text) in the paper. The main corrections in the paper and the point-by-point responses to the Referees’ comments are as following:

**#Review1**

**In this manuscript, Chen et al. mainly emphasize the need to understand the complex relationship between type 2 diabetes mellitus (T2DM) and osteoporosis, particularly concerning ferroptosis. The manuscript thoroughly explores the concept of ferroptosis, focusing on its role in DOP. Furthermore, iron-dependent lipid peroxidation, particularly impacting pancreatic β-cells, osteoblast, and osteoclast, contributes to Diabetic osteoporosis (DOP). Overall, the logic of this manuscript is clear, and the summary is robust and of high quality. In short, this work can be published in JTM after minor revision.**

**Response: we thank the reviewer for giving us opportunity to revise this manuscript. All the reviewer’s comments have been replied point-by -point.**

*****1: Overall, the manuscript conveys essential information, but English writing shows poor quality and requires further editing with the help of a professional service.*****

**We tried our best to imoprove the manuscript and made some changes to the manuscript. At the same time, we let editage.com to help polish our artical. These changes will not influence the content and framework of the paper. We appreciate for editors warm work earnestly and hope that the correction will meet with the approval.**

*****2: The author should summarize and discuss the***** *****therapeutic opportunities in this paper, which will significantly enrich this article. The author can reference a paper recently polished and named as***** *****Iron metabolism and ferroptosis in type 2 diabetes mellitus and complications: mechanisms and therapeutic opportunities*****

**We sincerely appreciate your valuable comments. We have carefully reviewed the literature and have added more relevant information on treatment opportunities in the final part of the revised draft. Of course, we have also drawn on the literature that you have recommended.**

*****3: I remember a paper that discussed the Mechanism of Ferroptosis and Its Role in Type 2 Diabetes Mellitus***** *****in J Diabeters Res journal 2021. It will be more suitable after being discussed in this manuscript.*****

**Thanks to your kind recommendation, we found this article in J Diabeters Res, issue 2021, and after careful reading, we have rearranged the mechanism of ferroptosis in T2DM and greatly enriched the revised version by adding relevant content on adipocyte metabolism and liver metabolism.**

**On page 13, Sha, W., Hu, F., Xi, Y., Chu, Y., & Bu, S. (2021). Mechanism of Ferroptosis and Its Role in Type 2 Diabetes Mellitus. Journal of Diabetes Research, 2021, 1–10. <https://doi.org/10.1155/2021/9999612>.**

*****4: A simple historical map of the development of ferroptosis will make it easier to understand for every reader.*****

**Based on your suggestions, we have created a comprehensive map that effectively illustrates the mechanism of ferroptosis. This map will undoubtedly help readers gain a better understanding of the full text.**

*****5: It will enrich this manuscript and help the author have a deeper understanding of the ferroptosis from a paper named as***** *****Ferroptosis and Traditional Chinese Medicine for Type 2 Diabetes Mellitus*****

**Thanks to your kind recommendation, we found this article. And this paper helped us and enriched our article, including therapeutic opportunities and comprehensive ferroptosis map.**

**#Review2**

**In this manuscript, ferroptosis in osteoporosis, diabetes (mainly T2D), and DOP was separately expounded and summarized. It may help to discover new targets for the treatment of DOP based on ferroptosis. However, there were some issues in the manuscript.**

*****In 1.*** *Introduction part, the relationship between T2D and diabetes should be explained. And why it was focused on T2D should be analyzed. In the first paragraph, there was no introduction on the epidemiology of osteoporosis. The relationship linking diabetes, osteoporosis and DOP should be elucidated more, not only some epidemiological data.***

***In the second paragraph, more action mechanisms of DOP should be supplemented, and it helps to go to the "ferroptosis" naturally. In the last paragraph, more evidences on the relation of type 2 diabetic osteoporosis and ferroptosis should be supplied and well written.***

**We would like to express our immense gratitude to the reviewers for their incredibly thorough review.** We think all the above suggestions are good additions to the the introduction part***.***We have **revised** the introduction part based on the reviewers' suggestions. Firstly, more emphasis on T2DM is mainly based on the literature research finding that DOP has become one of the serious complications of T2DM.

Based on a large number of domestic and overseas research studies, we enriched the epidemiology of DOP, and at the same time, we updated the relevant prevalence data of DM according to the 10th edition of the IDF Diabetes Atlas. Regarding the relationship between T2DM, DOP and DOP we also conducted a literature study to discover the effect of diabetic microenvironment on osteoporotic diseases (added it in the second paragraph).

*****In 2. Ferroptosis part, it was too long and it should be concise and to the point.*****

**We have concised the section and hoped that the map of ferroptosis would help reader understand the mechanism better.**

*****In 3. Ferroptosis in OP part, "ferroptosis in bone marrow mesenchymal stem cells" should be supplied in addition to OBs and OCs.*****

Thank you for your careful reading and honest feedback! We have added a section of ferroptosis in bone marrow mesenchymal stem cells to better understand the effects of ferroptosis on the bone microenvironment.

*****In 4.Ferroptosis in DM part, it actually was T2D, subheading should be exact. And some related cells should be listed such as β-cells, hepatocytes, adipocyte and others. More characteristics of T2D should be reflected.*****

**Thank you for your reminder. We have made the necessary corrections in the corresponding section.**

*****In 5. Ferroptosis in T2DOP part, it was the most important part of the manuscript, which should be well written, analyzed and included some related contents in above 2, 3, 4 part.*****

**Thank you for the reminder. The mechanism of ferroptosis in T2DM and greatly enriched the revised version by adding relevant content on bone marrow mesenchymal stem cells, adipocyte metabolism and liver metabolism. But the adipocyte/liver metabolism might not related contents T2DOP. The bone marrow mesenchymal stem cells contents have been discussed in the revision。**
