# Supplementary material for: Type 2 diabetic mellitus related osteoporosis: focusing on ferroptosis
Source: J Transl Med. 2024 Apr 30;22:409. doi: 10.1186/s12967-024-05191-x (PMC11064363; doi:10.1186/s12967-024-05191-x)
Supplement: Supplementary file 5 — Supplementary Material 5 [file 12967_2024_5191_MOESM5_ESM.docx]

cover letter

Dear Dr Editor,

I hope this message finds you well. Please allow us to submit our manuscript entitled

"Type 2 diabetic Mellitus related Osteoporosis: focusing on ferroptosis" for your esteemed consideration in "Journal of Translational Medicine".

This concise review highlights the urgent need to understand the intricate relationship

between type 2 diabetes mellitus and osteoporosis, with particular emphasis on the

role of ferroptosis. The primary aim of this review is to elucidate the pathogenesis of

ferroptosis in diabetic osteoporosis and to provide an overview of future research

aimed at intervening in the field of cellular ferroptosis.

All contributing authors have thoroughly reviewed the manuscript and unanimously

agreed to submit it to your esteemed journal.

We sincerely appreciate your time and consideration in reviewing our submission and

look forward to receiving feedback from the esteemed reviewers. Should you require

any further information or clarification, please do not hesitate to contact me.

Thank you for considering our work.

Yours sincerely,

Yili Chen

Corresponding author:

Luyuan Chen (cly900115@163.com)

Haizhou Wang [(bigbigwolf1@163.com)](mailto:(bigbigwolf1@163.com))

**Declaration of competing interests**

The authors declare that they have no known competing financial interests or personal

relationships that could have influenced the work reported in this paper
